# Does Sponsorship Promote Equity in Career Advancement in Academic Medicine? A Scoping Review

**DOI:** 10.1007/s11606-023-08542-4

**Published:** 2023-12-06

**Authors:** Rachel Schwartz, Mia F. Williams, Mitchell D. Feldman

**Affiliations:** 1 Department of Anesthesia and Perioperative Care, University of California, San Francisco, San Francisco, CA USA; 2 Division of General Internal Medicine, Department of Medicine, University of California, San Francisco, San Francisco, CA USA

**Keywords:** gender, underrepresented, mentoring, leadership, sponsorship.

## Abstract

Sponsorship describes a set of actions wherein an influential champion (*sponsor*) uses their position to actively support a colleague’s career by helping them gain visibility, recognition, and/or positions. There is growing awareness of the importance of sponsorship for career advancement in academic medicine, particularly for women and those who are historically underrepresented and excluded in medicine (UIM). This scoping review examines the current landscape of evidence, and knowledge gaps, on sponsorship as it relates to career advancement in academic medicine for women and UIM faculty*.* We searched peer-reviewed literature in PubMed, Embase, and Web of Science (WoS) over the past 50 years (from 1973 through July 2023). Sixteen studies were included in the final review. We found relative consensus on sponsorship definition and value to career advancement. Heterogeneity in study design limited our ability to directly compare study outcomes. All included studies focused on gender differences in sponsorship: two of four quantitative studies found men were more likely to receive sponsorship, one reported no gender differences, and one was insufficiently powered. All but one of the qualitative studies reported gender differences, with women less likely to access or be identified for sponsorship. The mixed-methods studies suggested sponsorship may vary by career stage. Only two studies analyzed sponsorship for UIM populations. The existing data are inconclusive regarding best ways to measure and assess sponsorship, what institutional support (e.g., structured programs, formal recognition, or incentives for sponsorship) should look like, and at what career stage sponsorship is most important. Addressing this knowledge gap will be critically important for understanding what sponsorship best practices, if any, should be used to promote equity in career advancement in academic medicine. We advocate for commitment at the institutional and national levels to develop new infrastructure for transparently and equitably supporting women and UIM in career advancement.

## BACKGROUND

Achieving equity in career advancement for women and those historically underrepresented and excluded in medicine (UIM) is a critical concern in academic medicine. Sponsorship has emerged as a key new approach to address and ameliorate the persistent disparities that confront women and UIM faculty in career advancement. ^1–4^ Sponsorship, in this context, refers to an individual in an influential position who advocates for, guides, and provides opportunities to advance a colleague’s career. ^5^ This form of support extends beyond and is distinct from traditional mentorship, encompassing active endorsement, networking assistance, and strategic exposure to influential circles. ^6–8^

The concept of sponsorship as a vehicle for achieving equity has garnered increasing attention in academia. Some have argued that mentorship, while essential, may fall short in addressing systemic inequities, perhaps because mentorship focuses on the professional development of the mentee but is not directly dedicated to career advancement. ^9^ Sponsorship, on the other hand, takes a proactive approach by directly connecting their proteges or sponsees with opportunities for skill development, high-visibility projects, and access to decision-making forums (what is colloquially known as a “seat at the table”). Successful implementation of sponsorship in the corporate sector has provided promising insights into the potential of sponsorship to mitigate inequities. ^8, 10^ Corporate data reveals that sponsorship can lead to increased representation of traditionally excluded groups in leadership positions, breaking the cycle of traditional hierarchical imbalances. ^6–8, 11, 12^ Furthermore, in the business world, sponsorship has shown to be particularly effective for women and minority individuals, as it aids in overcoming structural barriers by ensuring direct exposure to influential stakeholders. ^6–8, 13^ These findings underscore the need for a review of sponsorship’s applicability within the unique context of academic medicine.^1^

Given the preponderance of commentaries from the health sciences and business literature on the value of sponsorship for career ^14–18^ and leadership ^13, 19^ advancement, it is clear that sponsorship is both distinct from and adds unique value to mentorship. Our review is aimed at examining the current evidence, and knowledge gaps, on sponsorship as it relates to career advancement in academic medicine for women and UIM faculty*.* We aim to illuminate the landscape of sponsorship within academic medicine and to provide evidence-based recommendations for fostering equitable career advancement for all.

## METHODS

As the goal of this review was to identify the current state of the literature on sponsorship as it relates to career advancement for women and UIM faculty, we chose to pursue a scoping review rather than a systematic review. ^20^

### Information Sources

Two authors (RS, MFW) searched PubMed, Embase, and Web of Science (Wos) for peer-reviewed studies published over the last 50 years (between 1973 and July 2023). Search terms for the three databases appear in Table 1.

**Table 1:** Database Search Terms

Database	Search terms
PubMed	(faculty[tiab] OR career[tiab]) AND sponsor*[tiab] AND (diversity OR equity OR underrepresented OR marginalized OR minority OR minorities OR female OR women)
Embase	(faculty:ab,ti OR career:ab,ti) AND sponsor*:ab,ti AND ('diversity'/exp OR diversity OR 'equity'/exp OR equity OR underrepresented OR marginalized OR minority OR minorities OR 'female'/exp OR female OR 'women'/exp OR women) AND ([article]/lim OR [article in press]/lim OR [review]/lim OR [preprint]/lim) AND [embase]/lim
Web of Science	Abstract search: (faculty OR career) AND sponsor* All fields search: diversity OR equity OR underrepresented OR marginalized OR minority OR minorities OR female OR women

The combined search yielded 924 results, of which 318 were duplicates. The two first authors both screened the remaining 606 abstracts and resolved any conflicts through discussion.

### Eligibility Criteria

The PICOS chart (Table 2) provides an overview of eligibility criteria. Sponsorship needed to be mentioned in the Results section of the manuscript in order to qualify as producing new data (i.e., studies that mentioned sponsorship in the Introduction and the Discussion but did not produce sponsorship data in the Results were excluded). Other inclusion criteria were that “sponsorship” had to refer to sponsorship behaviors that aligned with the definition of sponsorship described in our introduction. Articles that exclusively focused on financial sponsorship were excluded.

**Table 2:** PICOS, and Inclusion and Exclusion Criteria Applied to Database Search

PICOS	INCLUSION CRITERIA	EXCLUSION CRITERIA
Population	• Academic medicine • Career stage: Graduate level or above (e.g., residency level on for medical trainees, graduate doctoral program on for PhDs) • Women or UIM focus	• Health professions (dentistry, nursing, etc.) • Faculty and trainees in STEM
Intervention	• Sponsorship focus • Career advancement focus	•Wrong definition of sponsorship (e.g., financial)
Comparison	•Men and Women’s sponsorship experiences • UIM sponsorship experiences relative to non-UIM • Prevalence of sponsorship	
Outcome	• To identify the presence and value of sponsorship on women and UIM career advancement	
Study Design	• Articles published from 1973 until July 2023 • Published in English • Databases: PubMed, Embase, WoS • Peer-reviewed • Cross-sectional survey, qualitative interviews, mixed-methods research	• Commentary, editorial, or perspective pieces • Review articles

### Study Selection

Citations and abstracts were uploaded to Rayyan Ai. ^21^ The first two authors independently screened all titles and abstracts. Conflicts about inclusion decisions were resolved through discussion and consensus with all authors through an iterative process.

### Data Extraction

**Figure 1:**
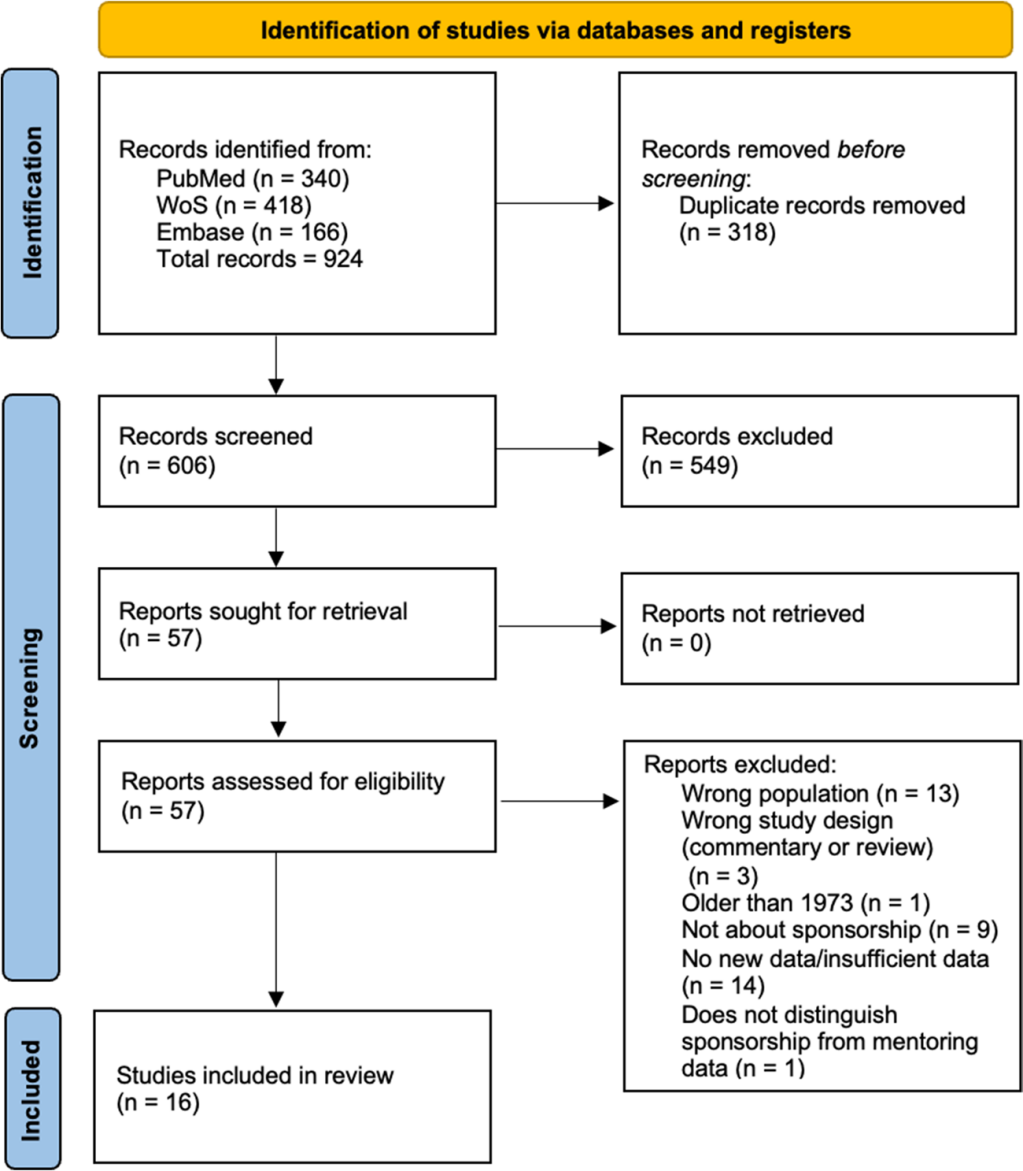
**PRISMA flow diagram.**

The two first authors independently reviewed all of the full-text manuscripts, extracting data on study objective, methods, participants, results, limitations, and definition of sponsorship and career advancement for each article. All authors reconciled any differences relating to inclusion criteria through discussion and mutual consensus. Ultimately, 16 papers met inclusion criteria (Fig. 1).

The authors adhered to the Preferred Reporting Items for Systematic reviews and Meta-Analyses extension for Scoping Reviews (PRISMA-ScR) guidelines. ^22^ As this was a scoping and not a systematic review, we present the existing evidence, regardless of quality, in order to understand the current state of the field rather than to compare the evidence between studies or conclusively answer a research question. ^23^

## RESULTS

According to the eligibility criteria, 549 articles were excluded at the abstract screening phase. Fifty-seven articles proceeded to full-text review. Sixteen were ultimately included in the final synthesis (Table 3).

**Table 3:** Included Studies

Study design	Article	Participants	Career stage(s)	Outcomes specific to sponsorship
Quantitative	Lalani et al. (2018) ^24^	221 radiation oncology faculty (34% women)	Early career	• 61% received at least one act of sponsorship • No significant gender differences
	Patton et al. (2017) ^25^	995 recipients of NIH K08 and K23 grants (461 women) who remained in academia	Undisclosed	• Sponsorship significantly associated with success • Males significantly more likely to report receiving sponsorship
	Pololi et al. (2016) ^26^	1520 faculty (510 women) across four institutions in US and UK	Undisclosed	• Statistical trend (p = .07) towards male faculty receiving more sponsorship than female faculty
	Grass and Latal (2022) ^27^	38 current and alumni participants (31 female) of two medical faculties in Switzerland	Undisclosed	• 34% did not have a sponsor • 10.5% unsure of if they had a sponsor • 13% unfamiliar with sponsorship • 29% of sample actively sought out a sponsor • 53% reported never seeking a sponsor • Gender comparison absent due to 80% female participants • Participants highlighted role of sponsorship in “breaking the glass [ceiling]” for women • Reported successful careers are not possible without sponsorship • More participants had male sponsors • Sponsorship seen as most important during the mid-career phase
	Seehusen et al. (2021) ^28^	193 family medicine department chairs; of 96 reporting gender, 35 were women	Late career	• Inadequately powered to detect gender differences • Only 25% had received training in sponsorship • Training associated with more frequent sponsoring
Qualitative	Ayyala et al. (2019) ^4^	12 sponsors (0 women) and 11 proteges* (6 women)	Across career stages	• Sponsorship as episodic and focused on specific opportunities • Effective sponsors were well-connected and established • Effective proteges rose to the task and were loyal • Sponsorship relationship involved trust, respect, weighing risks • Sponsorship seen as critical for career advancement • Women less likely to seek out sponsorship but in need of that support
	Basile et al. (2023) ^29^	26 women leaders in anesthesiology	Late career; two retired	• Sponsorship seen as the dominant pathway for women to move into leadership roles • Participants noted men have opportunities for sponsorship earlier in their careers than women • Men more often sponsor men
	Farlow et al. (2023) ^30^	12 women otolaryngologists	Mid- and late career	• Women disadvantaged in career advancement due to lack of access to sponsors and lack of negotiation training • Few participants sought out sponsorship • Highlighted the need for cultivating communities of women to facilitate awareness of opportunities • Most participants reported sponsoring more women than men • Called for institutions to implement equitable, transparent policies and programs to enhance sponsorship for women
	Guptill et al. (2018) ^31^	22 women leaders in emergency medicine	Late career	• Women described seeking sponsors for career advancement • Noted not enough sponsors championing women
	Hilsabeck (2018) ^32^	20 neuropsychologists (10 women)	Mid- and late career	• None had received formal training in sponsoring • On average, each person sponsored 40 others • 30% said they were approached by all their sponsees; 20% said it was mutually agreed • No significant gender differences found on number of those sponsored or receiving sponsorship • Small effects suggested for: women sponsoring greater number than men and men having more sponsors than women
	Hobgood and Draucker (2022) ^33^	37 current or former emergency medicine department chairs (19 women)	Late career	• Gender differences in career advancement: men advanced with the sponsorship of senior leaders; women advanced through own efforts • Men reported receiving leadership validation, and nomination for leadership opportunities that “fast-tracked” their careers
	Levine et al. (2021) ^34^	12 sponsors (0 women) and 11 proteges (6 women), 4 UIM	Across career stages	• Women less likely to seek out, or be identified for, sponsorship • Women and UIM were believed to particularly benefit from the credibility and access to networks and resources sponsorship confers • Noted disconnect between sponsorship and professional norms such as transparency, merit-based advancement, and fairness
	Mahendran et al. (2022) ^35^	14 women surgeons, 11 women residents, 10 women fourth-year med students; only reporting data from faculty here	Across career stages	• Sponsorship seen to benefit women “at every career stage” • Faculty reported difficulty accessing sponsorship • Female faculty felt leadership positions difficult to acquire and that this affected their ability to sponsor others • Highlighted the need for future research on sponsorship for UIM populations
Mixed-Methods	Manne-Goehler et al. (2020) ^36^	52 focus group participants (37 women); 790 survey respondents (458 women); all faculty in infectious disease	Across career stages	• Female full professors significantly more likely to report having received sponsorship than male full professors • No gender differences reported in sponsorship for other career stages • The importance of sponsorship for advancement was strongly endorsed
	Stephenson et al. (2022) ^37^	293 female physicians, physician leaders, physician faculty, and researchers	Undisclosed	• Lack of sponsorship was a significant negative predictor of satisfaction: for every one unit increase in lack of sponsorship, satisfaction decreased by 0.28 • 89% of the women reported downplaying accomplishments, and 87% reported being cautious when self-promoting • 70% reported having to work harder than male colleagues for the same credibility
	Williams et al. (2023) ^5^	903 faculty (477 women; 95 UIM)	Across career stages	• 55% perceived women receive less sponsorship than men and 46% perceived UIM receive less sponsorship than peers • Early- and mid-career faculty were more familiar with sponsorship than late-career faculty, and higher among women than men and among UIM compared to non-UIM • 76% reported having a personal sponsor

^*^In this table, the terms protégé and sponsee are used interchangeably, based on the terms used in the manuscript described

There was heterogeneity in study design, medical specialty, and career stage focus of the included studies. Five were purely quantitative, with one focusing on tool validation; the others used cross-sectional, often retrospective surveys. Eight were qualitative, involving semi-structured interviews. Three involved mixed-methods evaluation that included either focus groups or open-ended survey data that underwent thematic analysis.

### Definition of Sponsorship

All included studies provided a description of sponsorship or acts of sponsorship; however, the definition was not consistently stated at the outset. There was general consensus on three elements unique to sponsorship: (1) Sponsorship is specifically focused on the *provision of career advancement opportunities*; (2) sponsorship *requires the sponsor to hold a position of power or influence*; (3) *sponsorship involves advocacy by the sponsor for the sponsee.* Two articles additionally highlighted the role of a sponsor in *protecting their sponsees. *^5, 31^

Studies varied in their description of the nature of the sponsorship relationship, with some calling it “episodic” or “transactional,” ^29, 35^ while others focused on more longitudinal elements of the relationship, noting that a sponsee “is distinguished by loyalty to the sponsor” ^27^ and that sponsorship requires a “reputational risk” to the sponsor through the public commitment of an individual whose talents they are promoting. ^35^ Mahendran et al. ^35^ and Hilsabeck ^32^ highlighted the bi-directional nature of the sponsorship relationship, with both noting the intrinsic satisfaction of seeing a protégé succeed. A precursor to sponsorship appears to be sufficient interpersonal connection for a sponsee’s talent to be recognized. Table 4 provides an overview of sponsorship definitions.

**Table 4:** Sponsorship Definitions

Element 1: Provision of career advancement opportunities	Element 2: Sponsor holding position of power/influence	Element 3: Advocacy for the sponsee
• “applied as a deliberate strategy for career advancement of the sponsee and is critical for high level advancement” ^27^ • “the explicit goal of sponsorship is career advancement for the sponsored” ^28^	• “faculty in a position of influence and power (with access to networks and resources)” ^4^ • “A sponsor is usually someone with clout” ^32^ • “The definition of sponsorship highlights the power and influence of the sponsor” ^27^	• “advocacy on behalf of a high-potential junior person by powerful senior leaders” ^25^ • “highly placed individual…who influences decisions regarding appointments to committees, promotions, and awards” ^36^ • “utilization of power and influence to advocate for an individual” ^30^

### Overview of Sponsorship Themes

The data extracted from the analyzed studies revealed salient themes relating to career sponsorship in academic medicine. The most common themes highlighted were the following: significant impact of sponsorship on career success and breaking barriers; enhanced impact on career for women; differences in receipt and delivery of sponsorship for women and UIM individuals; and lack of awareness of sponsorship and how best to seek sponsorship.

### Effects of Sponsorship on Career Advancement

Three studies provided definitions or criteria for career advancement. While the others did not explicitly define career advancement, per our inclusion criteria, all mentioned career advancement in association with sponsorship, as either a goal or outcome (Table 5). Ten studies reported that sponsorship was associated with career advancement, often as a critical component ^4, 5, 25, 27–29, 33–36^.

**Table 5:** Included Studies’ Definition of Career Advancement and Support for Sponsorship’s Role

Definition of career advancement	Support of sponsorship’s role in career advancement
• Achieving at least one of the following: (1) being PI on an R01 or total grant funding over 1 million dollars; (2) publishing 35 or more peer-reviewed papers; ( 3) appointment as dean, department chair, or division chief ^25^ • Advancement to full professor ^29, 36^ • Progress to top leadership roles ^37^	Quantitative and mixed-methods studies: • Percent of participants that felt sponsorship played a significant role in their professional development: 60% ^5^ across 832 faculty from diverse fields; 50% ^28^ of 105 family medicine department chairs • Percent of family medicine department chairs reporting sponsorship as the tool that played the largest role in ascension to their leadership career: 15% ^28^
	Qualitative studies: • “sponsorship is critical to career advancement” ^4^ • “sponsorship was the dominant pathway for women moving into leadership roles” ^29^ • “Both sponsors and protégés reported that sponsorship was critical to high-level advancement. Women and UIM faculty were believed to benefit specifically from the external credibility and access to networks and resources that sponsorship provides” ^34^

### Gender Differences in Sponsorship

Two of the four quantitative studies that reported on gender differences found that men were more likely to report receiving sponsorship ^25^ or found a trend towards men receiving more sponsorship. ^26^ The other two either found no difference ^24^ or were insufficiently powered to draw conclusions. ^28^

Of the qualitative studies in our review, women reported a lack of access to sponsorship ^30, 35^ compared to men who received more sponsorship ^33^, and earlier in their careers. ^29^ It was noted that men are more likely to sponsor men ^29^, and women are less likely to seek out or be identified for sponsorship. ^4, 34^ Only one qualitative study reported no gender differences. ^32^

Finally, the mixed-methods studies that included a gender comparison offered a more nuanced understanding of the relationship between gender, career-stage, and sponsorship. In one study, women full professors were significantly more likely to report having received sponsorship to arrive at the leadership position than their male peers ^36^, while a separate study found no gender differences at the same stage. ^5^ At the early-career stage, one study found distinct differences in patterns of sponsorship between the early- and mid-career stages, with women receiving more sponsorship than their male peers at the assistant professor level; however, women were significantly less likely than men to receive sponsorship at the associate level. ^5^

### Sponsorship Value for UIM

Two studies, Levine et al. ^34^ and Williams et al. ^5^, analyzed the value of sponsorship for UIM individuals. Levine et al. note that among UIM faculty interviewed, there was a sense that sponsorship offered them enhanced external credibility and privileged access to influential networks. Williams et al. delved further into sponsorship experiences and perceptions using a quantitative approach. In that study, the experience of UIM faculty varied by academic rank. Familiarity and receipt of sponsorship was higher among junior UIM faculty compared to their non-UIM peers, while the inverse was true at the associate and full professor levels. Regarding delivery of sponsorship, more full-professor faculty reported serving as sponsors than their non-UIM peers; the same was not true at the assistant level. They also found that among the total faculty sample, a significant proportion perceived inequities in the receipt of sponsorship for UIM faculty members.

### Received Training on Sponsorship

Two studies examined the prevalence of sponsorship training. Seehusen et al. ^28^ reported 26% of their sample had received training on sponsorship and 54% of their sample frequently used sponsorship as a tool for faculty development. Hilsabeck ^32^ reported that none of their participants had received formal training on sponsorship. However, they identified the most valued and the least desirable sponsee characteristics.

### Critical Junctures and Career Stages

Four of the included studies did not identify the career stage of their participants. Of the remaining 12 studies, one focused on early-career faculty ^24^, two focused on mid- and late-career faculty ^30, 32^, four on late-career faculty ^28, 31, 33^ (including some retired faculty ^29^), and five included participants from across career stages. ^4, 5, 34–36^ One study that did not disclose the career stage of participants reported that respondents considered sponsorship most important at the mid-career stage, and that the importance of sponsorship lasted through late-career stages. ^27^ One study noted that as faculty advanced in their career, access to sponsorship became more limited. ^35^ Across studies, a theme that emerged was the importance of having a sponsor who was in a position of power, often someone at a late-career stage.

### Included Studies’ Guidance for Addressing Inequities

**Figure 2:**
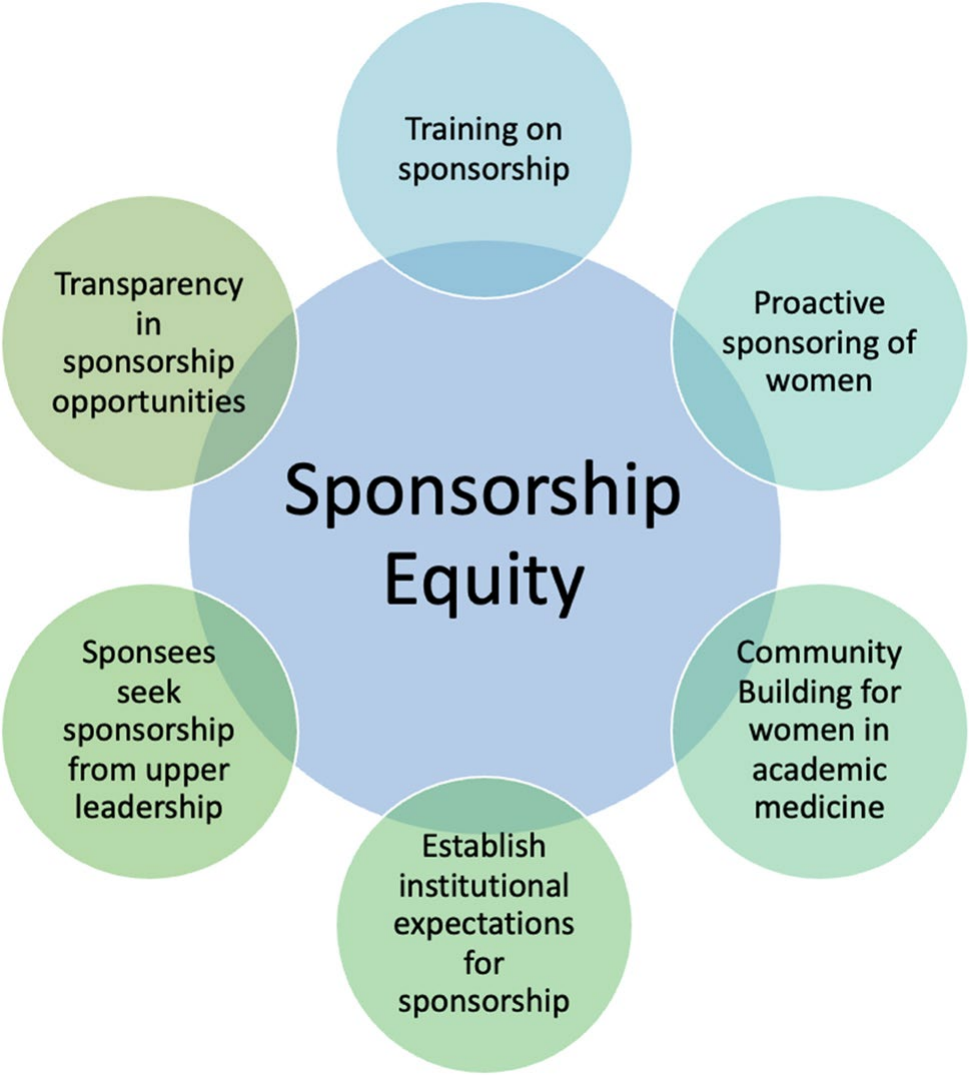
**Studies’ proposed interventions for addressing inequity in sponsorship.**

The included studies proposed multiple avenues for addressing sponsorship inequity (Fig. 2). The most highly endorsed approach was establishing institutional expectations for sponsorship, including metrics for assessing it and expectations around promotion of women. ^5, 27, 30, 34, 37^ The other most prevalent proposed intervention was implementing sponsorship training programs. ^5, 28, 31, 32^ Studies advocated for transparency in sponsorship opportunities for women, ^5, 30, 33^ community building for women in academic medicine to raise awareness of available opportunities, ^24^ encouraging proactive sponsorship of women, ^31, 36^ and having sponsees approach upper leadership to ask for sponsorship. ^4, 25^

## DISCUSSION

In this scoping review, we examined the current literature on sponsorship as it relates to career advancement for women and those underrepresented in medicine (UIM). We found general consensus on the definition of sponsorship and the value of sponsorship for career advancement. Studies highlighted the burgeoning awareness, and importance, of sponsorship ^5, 35^ and noted that the lack of awareness of sponsorship limits the ability to seek it. ^35^ There appears to be a lack of training in sponsorship, despite the recognized importance for faculty career advancement.

Our review highlights the need for more equitable and pro-active sponsorship of women in medicine in order to achieve gender diversity in career advancement and leadership representation. The data are inconclusive regarding the most important career stage(s) for sponsorship intervention, but the patterns observed in our review suggest differences in both the prevalence and impact of sponsorship for women. Notably, our scoping review did not yield any articles that provided descriptions of formal sponsorship programs or intensity/dose of sponsorship.

Our review found a limited landscape regarding the influence of sponsorship on UIM cohorts, as only two of 16 studies directly addressed this phenomenon. A diverse workforce is critically important for improving patient care, ^38^ creative work, ^39^ and scholarship. ^40^ Mentorship helps to support increased diversity, ^41–43^ but the goal goes beyond increasing diversity to supporting more equitable opportunities for career advancement, which many of the articles in the current review suggest requires, or at the very least, is significantly facilitated by sponsorship. Future sponsorship research must include UIM individuals and proactively create more opportunities for UIM to access sponsorship in order to achieve the goal of more diversity in leadership.

### Different Levers for Addressing Inequity

As seen in Figure 2, opportunities for sponsorship interventions to promote equity in career advancement exist at multiple levels. At the *national level*, more effort can be made towards community building for women (and ideally, UIM) and facilitating increased interaction with leaders to allow for networking and proactive sharing of available career-promoting opportunities. At the *institutional level*, new protocols can be established setting institutional expectations for sponsorship as has been done for mentorship at some academic health centers. ^44^ Sponsorship training programs should be developed to support proactive sponsorship of women and UIM faculty. Finally, new practices should be established to promote transparency in available leadership opportunities that help to support more equity in career advancement.

### Re-examining the Sponsorship Relationship

There were differing opinions among studies as to the nature of the sponsorship relationship. Multiple studies referred to sponsorship as “episodic” or “transactional”; however, it is evident from the description of effective sponsorship behaviors that acts of sponsorship rely on the sponsor recognizing the abilities and potential of a sponsee and vouching for the sponsee’s professional abilities. This type of recognition requires more than superficial knowledge of a person, and potential risk to the sponsor’s reputation if the sponsee performs poorly. Several articles in our review highlighted the “loyalty” required of the sponsee, which also suggests an ongoing relationship and commitment, even if the frequency of contact may be low. More attention to the relational precursors to sponsorship is needed to understand how best to develop and foster sponsorship in academic medicine and to ensure it helps to promote more, not less, diversity of opportunity and leadership.

#### Limitations

Our findings are limited by a relative paucity of research on sponsorship; this may in part be due to the nature of sponsorship, which often happens behind closed doors, making it difficult to track and quantify. ^35^ Increasing awareness and actions of sponsorship requires educating those in a position to sponsor others about its value in providing career advancement opportunities, particularly for those who come from backgrounds that have been historically excluded from leadership positions. While most articles reported on the career stage of participants, the interaction between receipt of sponsorship and career stage was not routinely reported, limiting our ability to draw conclusions. Finally, this scoping review may not have captured original research that did not contain an abstract or include “sponsorship” in the title.

## CONCLUSION

Although sponsorship research is nascent and still evolving, the evidence presented here demonstrates that sponsorship matters for career advancement in academic medicine for women and those who have been historically underrepresented and excluded in medicine (UIM). Leaders must commit themselves to creating a culture of sponsorship, as many have for mentorship, in order to address the current lack of diversity in leadership and equitable support for career advancement for those faculty and trainees who have historically been excluded from these opportunities. We advocate for commitment at the institutional level for program support and training, and at the national level for funding to develop, implement, and assess the impact of sponsorship programs for women and UIM faculty in academic medicine. This process will require individuals in positions of influence to reflect on how they approach sponsorship vis a vis equity, and develop new infrastructure for equitably supporting women and UIM in career advancement. Such initiatives may include new, transparent practices for leadership opportunities, and developing new networks for proactively sponsoring those who our data show are unlikely to seek it for reasons unrelated to their competence and potential.

## Data Availability

Data are available from the corresponding author upon reasonable request.

## Footnotes

1 We defined “academic medicine” as including faculty and fellows in academic pursuits with broadly defined scholarship.
